# Patterns and Trends in Mortality Associated With and Due to Diabetes Mellitus in a Transitioning Region With 3.17 Million People: Observational Study

**DOI:** 10.2196/43687

**Published:** 2023-09-04

**Authors:** Xiaopan Li, Ru Liu, Yichen Chen, Yan Han, Qizhe Wang, Yaxin Xu, Jing Zhou, Sunfang Jiang

**Affiliations:** 1 Department of Health Management Center Zhongshan Hospital Fudan University Shanghai China; 2 Department of General Practice Zhongshan Hospital Fudan University Shanghai China; 3 Office of Scientific Research and Information Management Center for Disease Control and Prevention Pudong New Area Shanghai China; 4 School of Public Health Fudan University Shanghai China

**Keywords:** diabetes mellitus, mortality, years of life lost, multimorbidity, trend analysis, diabetes, disease, urbanization, aging, epidemiology

## Abstract

**Background:**

Diabetes mellitus (DM) imposes a significant disease burden in economically transitioning regions. Most transitioning regions share similar experience in urbanization processes. Shanghai’s Pudong district serves as a representative area of such regions.

**Objective:**

We aimed to assess the burden of and trends in DM mortality in Shanghai’s Pudong district and analyze the impact of aging and multimorbidity.

**Methods:**

A longitudinal, population-based study was conducted to analyze DM mortality in Pudong from 2005 to 2020. We used joinpoint regression to analyze epidemiological features and long-term trends in crude mortality rate (CMR), age-standardized mortality rate worldwide (ASMRW), and years of life lost (YLL). Furthermore, the decomposition method was used to evaluate the contribution of demographic and nondemographic factors associated with mortality.

**Results:**

There were 49,414 deaths among individuals with DM, including 15,512 deaths due to DM. The CMR and ASMRW were 109.55/10^5^ and 38.01/10^5^ person-years, respectively. Among the mortality associated with and due to DM, the total annual ASMRW increased by 3.65% (95% CI 3.25%-4.06%) and 1.38% (95% CI 0.74%-2.02%), respectively. Additionally, the total annual YLL rate increased by 4.98% (95% CI 3.92%-6.05%) and 2.68% (95% CI 1.34%-4.04%). The rates of YLL increase in persons aged 30 to 44 years (3.98%, 95% CI 0.32%-7.78%) and 45 to 59 years (4.31%, 95% CI 2.95%-5.69%) were followed by the increase in persons aged 80 years and older (10.53%, 95% CI 9.45%-11.62%) for deaths associated with DM. The annual CMR attributable to demographic factors increased by 41.9% (95% CI 17.73%-71.04%) and 36.72% (95% CI 16.69%-60.2%) for deaths associated with and due to DM, respectively. Hypertension, cerebrovascular disease, and ischemic heart disease were the top 3 comorbidities.

**Conclusions:**

Aging and multimorbidity played essential roles in changing the burden of DM in an urbanizing and transitioning region. There is an increasing disease burden among young and middle-aged people, emphasizing the need for greater attention to these groups. Health management is an emerging method that holds important implications for alleviating the future burden of DM.

## Introduction

Diabetes mellitus (DM) is a state of metabolic disorder characterized by hyperglycemia that can lead to damage in the heart, blood vessels, kidneys, eyes, and nerves [1]. Worldwide, the number of people with DM has quadrupled in the last 3 decades, and DM ranks ninth among the major causes of death [2]. In 2021, according to the *IDF Atlas, Tenth Edition*, an estimated 1 of 10 people aged 20 to 79 years had diabetes, and approximately 75% of people with DM lived in developing countries [3]. In China, the prevalence of DM increases with age; about 11.2% of people aged 18 years and older were living with DM from 2015 to 2017 [4]. Additionally, most patients with DM experience at least one complication that can be life-threatening [5]. DM is associated with an 8-year reduction in lifespan in the United States and has a negative effect on the quality of life, given the high incidence of complications. Approximately 12% of worldwide health expenditure in 2015 was spent on the treatment of DM and related complications [6].

With the strengthening of reforms and opening up, Shanghai’s Pudong district, with a population of 3.17 million, has become a center for trade, economic development, scientific advancements, and technological innovations; it has also witnessed notable improvements in public services, including enhanced social security coverage, long-term care, and education and health services. Pudong serves as a representative transitioning region that has experienced dramatic improvements in living standards. However, with these improvements, many risk factors for the incidence and progression of DM have increased, such as unhealthy eating habits, sedentary lifestyles, psychosocial stress, depression, and overweight or obesity [7]. The urbanization process in Pudong has substantially impacted the disease spectrum. Considering that other transitioning regions are likely to undergo similar urbanization processes, insights into the burden of DM and trends in DM-related mortality in Pudong can inform the management and planning of health care services in these regions in the future [8].

Without sufficient studies on disease-related and specific deaths, estimating the true burden of a disease is challenging [9]. It is also crucial to differentiate mortality associated with DM and due to DM. Such differentiation is essential for assessing the effectiveness of DM treatment and obtain valuable insights for policymakers in developing regions, enabling them to devise new health care strategies that address unmet needs. Therefore, our study aimed to assess the burden of DM mortality, examine its trends, and investigate the impacts of population aging and multimorbidity in Pudong, a transitional region undergoing urbanization.

## Methods

### Data Source

The study population consisted of residents of Pudong, Shanghai, with DM who had died and for whom data were available in the Mortality Registration System from 2005 to 2020. We selected Pudong as the study site for 3 reasons. First, Pudong encompasses both urban and rural subregions, covering an area of 1210.41 km^2^ with a population of 3.17 million [10]. Second, people older than 65 years account for 23.73% of the total registered population in Pudong. The large population base and significant proportion of hyper-aged residents allow for a more realistic evaluation of the epidemiological features of deaths associated with DM. Third, the gross domestic product in Pudong increased from RMB 210.8 billion (US $29.4 billion) in 2005 to RMB 1320.7 billion (US $184.2 billion) in 2020, with an average annual growth rate of 13.01% [11]. Therefore, with the fastest economic development among Shanghai districts, Pudong is recognized as a microcosm of China’s urbanization. The registration system is administered by the Public Security Bureau and the Statistics Bureau of Pudong. The system contains comprehensive population demographic characteristics, including age, sex, and cause of death. To maintain the integrity of the registration system, regular evaluations and data cleaning are carried out. DM was defined according to the *International Classification of Diseases, Tenth revision* (*ICD-10*) codes E10 to E14, and DM-related deaths were classified according to whether DM was a direct or indirect cause of death. More specifically, we screened all causes of death that included DM and defined them as death associated with DM. There were 4 listed direct causes of death on each certificate, one of which was used to define death due to DM. Death due to DM was determined by clinical doctors according to the patients’ situation and relevant examination. The causes of death were independently reviewed by rigorously trained clinicians from the local Center for Disease Control and Prevention (CDC) based on the actual circumstances of each patient.

### Ethics Approval

This surveillance protocol was approved by the Ethics Committee of the Shanghai Pudong CDC (2016-04-0586). The study did not involve any intervention among participants, and all data were maintained in strict confidentiality.

### *ICD-10* Codes

This study used the *ICD-10* codes E10 to E14 to identify causes of death related to DM. These codes include insulin-dependent DM (E10), non–insulin-dependent diabetes of the young (E11), malnutrition-related DM (E12), other specified DM (E13), and unspecified DM (E14). In addition, other diseases were classified as complications according to the *ICD-10*. To account for potential variation in data quality across different years, we followed a recommended method to evaluate data quality [12]. The method helps to improve the reliability of the results by defining and categorizing “garbage codes.”

### Statistical Analyses

Specific-cause mortality was calculated based on deaths with DM as the underlying cause of death (ie, deaths due to DM), while all-cause mortality was calculated based on deaths with DM among all causes of death (deaths associated with DM).

The crude mortality rate (CMR) and age-standardized mortality rate worldwide (ASMRW) for DM were calculated per 100,000 persons (/10^5^) and stratified by sex and year. We used the Poisson approximation method and the Mantel-Haenszel test to compare the CMR and ASMRW between sexes, respectively. Years of life lost (YLL) was used as a key measure to quantify the burden of disease and was calculated according to the original method described by Murray and Lopez that was detailed previously by Ye et al [13]. The YLL formula adopted by the World Health Organization (WHO) [14,15] was applied. Mortality rates were calculated for the following age groups: 0 to 4 years, 5 to 14 years, 15 to 29 years, 30 to 44 years, 45 to 59 years, 60 to 69 years, 70 to 79 years, and ≥80 years. Due to there being few DM-related deaths before the age of 30, trends in age-specific CMR, ASMRW, and YLL were calculated for the following age groups: 0 to 29 years, 30 to 44 years, 45 to 59 years, 60 to 69 years, 70 to 79 years, and ≥80 years.

Joinpoint regression, a popular approach for analyzing changes in trends, was used in this study. We modeled the time series using continuous linear segments and minimized the weighted sum of squared errors and the number of joinpoints using permutation tests [16]. We used the Joinpoint Regression Program (version 4.3.1.0; National Cancer Institute) to calculate trends in CMR, ASMRW, and YLL over time. The results were presented as the average annual percent change (AAPC) with corresponding 95% CI. Using the *z* test, we determined whether the annual percent change (APC) significantly differed from zero [17]. The difference decomposition method was used to quantify determinants of demographic and nondemographic factors. The rates of DM distribution in different groups were calculated and presented as percentages. In the results section, we report statistically significant (*P*<.05) changes as increases and decreases in AAPC and nonsignificant trends as stable. All statistical analyses were performed using SPSS (version 21.0; IBM Inc) and R (version 3.4.3; R Core Team). The significance level was set at *P*<.05.

## Results

### Baseline Characteristics

**Figure 1:**
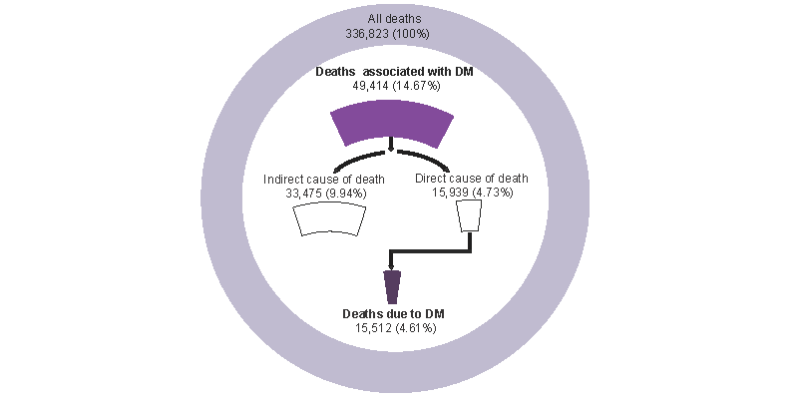
Flow chart of deaths selected from among all deaths in Pudong, Shanghai, from 2005 to 2020. DM: Diabetes mellitus.

In this study, we extracted 45,107,809 records at the person-year level from residents in Pudong from 2005 to 2020; during this period, 336,823 deaths occurred. Among these deaths, 49,414 were associated with DM, including 15,512 deaths due to DM (Figure 1 and Multimedia Appendix 1, Figure S1). Approximately equal representation of men and women was observed among deaths associated with DM, with women accounting for 51.01% of deaths. The average age at death was 77.83 (SD 10.72) years, and the median age at death was 79.83 years. The total CMR and ASMRW of deaths associated with DM were 109.55/10^5^ and 38.01/10^5^ person-years, respectively. Among men, the CMR and ASMRW were 107.54/10^5^ person-years and 43.84/10^5^ person-years, respectively; among women, the corresponding figures were 111.55/10^5^ person-years and 32.61/10^5^ person-years. Among the individuals who died due to DM, 53.59% were women; the CMR and ASMRW in women were 36.79/10^5^ person-years and 10.79/10^5^ person-years, respectively. The YLL due to premature death was 425,938.83 years, and the YLL rate was 944.27/10^5^ person-years for deaths associated with DM. The YLL and YLL rates in men (217,230.26 years and 964.92/10^5^ person-years) were higher than in women (208,708.56 years and 923.69/10^5^ person-years; Table 1). The YLL and YLL rates increased as age exceeded ≥80 years. The top 3 age groups in terms of YLL and YLL rates of deaths associated with DM were 70 to 79 years, ≥80 years, and 60 to 69 years, with YLL of 133,784.59, 125,400.22, and 100,335.50, respectively, and YLL rates of 4088.18/10^5^, 6668.12/10^5^, and 1614.79/10^5^, respectively; Multimedia Appendix 1, Table S1).

**Table 1:** Baseline characteristics of people who died of causes associated with and due to diabetes mellitus from 2005 to 2020.

Characteristic				Deaths, n (%)		Age at death (years)				CMR^a^, /10^5^		ASMRW^b^, /10^5^		YLL^c^, years		YLL rate, /10^5^
						Mean (SD)		Median (range)								
**Deaths associated with DM^d^**																
	**Gender**															
		Male	24,210 (48.99)		75.61 (11.19)		77.54 (17.22-105.90)		107.54		43.84		217,230.26		964.92	
		Female	25,204 (51.01)		79.96 (9.78)		81.69 (21.76-105.65)		111.55		32.61		208,708.56		923.69	
		Total	49,414 (100)		77.83 (10.72)		79.83 (17.22-105.90)		109.55		38.01		425,938.83		944.27	
**Top 3 comorbidities among deaths associated with DM**																
	Ischemic heart disease (*ICD-10*^e^ codes I20-I25)		9075 (18.37)		80.82 (9.40)		82.38 (27.15-105.19)		20.12		6.39		67,544.01		149.74	
	Cerebrovascular disease (*ICD-10* codes I60-I69)		8695 (17.60)		78.94 (9.69)		80.75 (30.70-105.65)		19.28		6.39		70,585.83		156.48	
	Lung cancer (*ICD-10* codes C33-C34)		2369 (4.79)		73.34 (9.37)		74.00 (32.70-98.59)		5.25		2.08		24,181.69		53.61	
**Deaths due to DM**																
	**Gender**															
		Male	7199 (46.41)		75.38 (12.01)		77.69 (20.36-101.86)		31.98		13.20		65,481.23		290.86	
		Female	8313 (53.59)		80.01 (10.26)		81.79 (21.76-105.07)		36.79		10.79		68,850.69		304.72	
		Total	15,512 (100)		77.86 (11.34)		80.13 (20.36-105.07)		34.39		11.98		134,331.92		297.80	
**Top 3 comorbidities among deaths due to DM**																
	Other diseases of the respiratory system (*ICD-10* codes J95-J99)		4062 (26.19)		79.53 (10.00)		81.37 (21.76-103.35)		9.01		2.96		32,091.78		71.14	
	Cerebrovascular disease (*ICD-10* codes I60-I69)		3841 (24.76)		78.39 (9.51)		79.95 (32.34-101.97)		8.52		2.87		31,956.80		70.85	
	Ischemic heart diseases (*ICD-10* codes I20-I25)		2953 (19.04)		79.86 (9.38)		81.39 (27.69-103.48)		6.55		2.13		23,054.70		51.11	

^a^CMR: crude mortality rate.

^b^ASMRW: age-standardized mortality rate worldwide.

^c^YLL: years of life lost.

^d^DM: diabetes mellitus.

^e^*ICD-10*: *International Classification of Diseases, Tenth revision*.

### Main Comorbidities

Among the deaths associated with DM, the top 3 comorbidities were ischemic heart disease (*ICD-10* codes I20-I25; 9075/49,414, 18.37%), cerebrovascular disease (*ICD-10* codes I60-I69; 8695/49,414, 17.6%) and lung cancer (*ICD-10* codes C33-C34; 2369/49,414, 4.79%). The YLL and YLL rates due to cerebrovascular disease were the highest (70,585.83 years and 156.48/10^5^, respectively), followed by ischemic heart disease (67,544.01 years and 149.74/10^5^; Table 1). The age at death, CMR, and ASMRW of these comorbidities are presented in Table 1.

The top 10 comorbidities among deaths associated with DM were the same for both men and women, with only the ranking differing; the sex-specific ranking is presented in Table 2. The ranking of comorbidities among deaths due to DM was also similar between men and women except for the ninth comorbidity, as shown in Table 3. On average, each person who died due to DM had 2.09 different comorbidities throughout their life. The disease spectrum of deaths associated with DM and due to DM is shown in Multimedia Appendix 1, Table S2.

**Table 2:** Top 10 comorbidities among men and women who died of causes associated with diabetes mellitus.

Type and cause of death (*ICD-10*^a^ code)	Men (n=24,210), n (%)	Women (n=25,204), n (%)
Diabetes mellitus (E10-14)	7199 (29.74)	8313 (32.98)
Cerebrovascular disease (I60-69)	4056 (16.75)	4639 (18.41)
Ischemic heart disease (I20-25)	3993 (16.49)	5082 (20.16)
Lung cancer (C33-34)	1669 (6.89)	700 (2.78)
Chronic lower respiratory disease (J40-47)	1459 (6.03)	419 (1.66)
Liver cancer (C22)	711 (2.94)	419 (1.66)
Colorectal cancer (C18-21)	582 (2.40)	473 (1.88)
Malignant neoplasm of the pancreas (C25)	549 (2.27)	503 (2.00)
Stomach cancer (N40-51)	530 (2.19)	357 (1.42)
Falls (W00-19)	256 (1.06)	326 (1.29)
Other diseases^b^	3206 (13.24)	4639 (18.41)

^a^*ICD-10*: *International Classification of Diseases, Tenth revision*.

^b^Other diseases are listed in Multimedia Appendix 1, Table S2.

**Table 3:** Top 10 comorbidities among men and women who died due to diabetes mellitus.

Type and cause of death (*ICD-10*^a^ code)	Men (n=15,419), n (%)	Women (n=16,958), n (%)
Hypertensive diseases (I10-15)	3520 (22.81)	4151 (24.48)
Other diseases of the respiratory system (J95-99)	2627 (17.03)	2627 (15.49)
Cerebrovascular disease (I60-69)	1869 (12.11)	2044 (12.05)
Ischemic heart disease (I20-25)	1343 (8.70)	1701 (10.03)
Heart disease (I05-52)	1307 (8.47)	1407 (8.30)
Renal failure (N17-19)	1290 (8.36)	1237 (7.29)
Chronic lower respiratory disease (J10-18)	740 (4.80)	651 (3.84)
Metabolic disorders (E70-90)	524 (3.40)	587 (3.46)
Ill-defined and unknown cause of mortality (R95-99)	362 (2.35)	352 (2.08)
General symptoms and signs (R50-69)	356 (2.31)	404 (2.38)
Other diseases^b^	1491 (9.66)	1733 (10.22)

^a^*ICD-10*: *International Classification of Diseases, Tenth revision*.

^b^Other diseases are listed in Multimedia Appendix 1, Table S2.

### DM-Specific Premature Death

**Figure 2:**
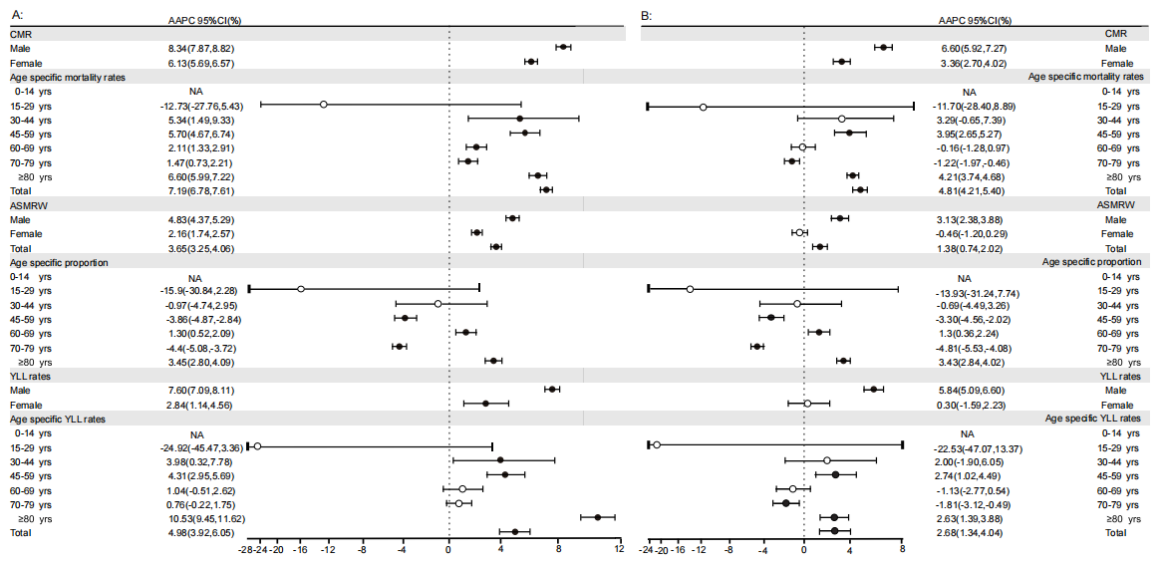
Trends in CMR, ASMRW, age-specific proportion of mortality, and YLL among people who died of causes associated with and due to DM according to sex and age group in Pudong, Shanghai, China, from 2005 to 2020. (A) Deaths associated with DM; (B) deaths due to DM. AAPC: average annual percent change; ASMRW: age-standardized mortality rate worldwide; CMR: crude mortality rate; DM: diabetes mellitus; N/A: not applicable; YLL: years of life lost.

The total YLL rate increased by 4.98% (95% CI 3.92%-6.05%; *P*<.001) per year and 2.68% (95% CI 1.34%-4.04%; *P*<.001) per year in terms of deaths associated with and deaths due to DM, respectively. The YLL rates of men and women who died due to DM or causes associated with DM increased (all *P*<.001), except for women (*P*=.74) who died due to DM. However, the YLL rates in people that died of causes associated with DM were inconsistent with increasing age; they increased by 3.98% (95% CI 0.32%-7.78%; *P*=.04), 4.31% (95% CI 2.95%-5.69%; *P*<.001), and 10.53% (95% CI 9.45%-11.62%; *P*<.001) per year in the people aged 30 to 44 years, 45 to 59 years, and ≥80 years, respectively. Regarding deaths due to DM, the YLL rate increased by 2.74% (95% CI 1.02%-4.49%; *P*=.004) and 2.63% (95% CI 1.39%-3.88%; *P*<.001) per year in people aged 45 to 59 years and ≥80 years, respectively, while they decreased by 1.81% (95% CI –3.12% to –0.49%; *P*=.01) per year in people aged 70 to 79 years. The YLL rates in the remaining age groups were stable (Figure 2).

### Trends in the DM Burden

The long-term trend for total CMR among people who died of causes associated with DM or due to DM increased by 7.19% and 4.81%, respectively. In terms of deaths associated with DM, the CMR also differed by sex (all *P*<.05) and by age over 30 years (all *P*<.05). For deaths due to DM, people aged 30 to 44 years (*P*=.09) and 60 to 69 years (*P*=.76) did not show significant differences in the CMR. The CMR of different age groups among people who died of causes associated with and due to DM are presented in Multimedia Appendix 1, Table S1. The tendencies of modeled CMR, ASMRW, and YLL rates according to sex and age group are shown in Figure 2. The observed CMR, ASMRW, and YLL rates are shown in Multimedia Appendix 1, Tables S3-S6, and the age-specific proportions of death are shown in Multimedia Appendix 1, Table S7. The ASMRW significantly increased among people who died of causes associated with and due to DM, with increases of 3.65% (male AAPC=4.83%; *P*<.001; female AAPC=2.16%; *P*<.001) and 1.38% (male AAPC=3.13%; *P*<.001; female AAPC=–0.46%; *P*=.21), respectively.

The proportion of deaths associated with DM and deaths due to DM varied across age groups; the proportion increased in people aged 60 to 69 years (1.3%, 95% CI 0.52%-2.09%; *P*=.003; 1.3%, 95% CI 0.36%-2.24%; *P*=.009, respectively) and ≥80 years (3.45%, 95% CI 2.8%-4.09%; *P*<.001; 3.43%, 95% CI 2.84%-4.02%; *P*<.001, respectively). However, for deaths associated with DM, the proportion of deaths decreased by 3.86% (95% CI –4.87% to –2.84%; *P*<.001) and 4.4% (95% CI –5.08% to –3.72%; *P*<.001) in people aged 45 to 59 years and 70 to 79 years, respectively. There were no significant changes in the proportion of deaths among people younger than 45 years (all *P*>.05; Figure 2).

### Quantitative Impacts of Demographic and Nondemographic Factors on Mortality Rates

Among people who died of causes associated with DM, the CMR attributable to nondemographic factors increased in men (AAPC=17.24%; *P*<.001), women (AAPC=24.16%; *P*=.002) and the total population (AAPC=17.82%; *P*<.001); the CMR attributable to demographic factors also increased by 42.53%, 41.47%, and 41.9% in these groups, respectively, from 2006 to 2020 (all *P*=.001). In terms of deaths due to DM, the CMR caused by nondemographic factors increased in women (AAPC=22.26%; *P*<.001) and the total population (AAPC=20.38%; *P*=.002); there were no significant changes in men (*P*=.10). In contrast, the CMR caused by demographic factors increased in men (AAPC=36.11%; *P*=.001), women (AAPC=37.5%; *P*<.001), and the total population (AAPC=36.72%; *P*<.001). The trends for increased CMR caused by demographic and nondemographic factors relative to the CMR for DM in 2005 are shown in Multimedia Appendix 1, Figure S2. The increased CMR caused by demographic and nondemographic factors is shown in Multimedia Appendix 1, Table S8.

## Discussion

### Principal Findings

DM and its complications have made considerable contributions to global mortality and disability burdens, particularly in regions undergoing rapid epidemiological transitions [1]. Several studies have demonstrated a decline in DM mortality in developed regions, such as the United States [18], Canada, United Kingdom [19], and Hong Kong [20]. Over the past 40 years of reform and opening up, Pudong has experienced the fastest modernization in China and serves as a representative area for studying the effects of urbanization. In this study, we used population-level data to provide a comprehensive overview of the secular trends in mortality associated with and due to DM among people in Pudong, Shanghai, from 2005 to 2020. We found that DM mortality persistently increased with age, despite annual fluctuations. The magnitude of the increase in DM mortality was similar for both men and women, with more quickly increasing rates of death associated with DM observed for people aged 30 to 44 years and 45 to 59 years. The top 3 comorbidities contributing to deaths associated with and due to DM were hypertension, cerebrovascular disease, and ischemic heart diseases. In addition, population aging and nondemographic factors also played an important role in the burden of DM.

The total CMR trend of death associated with DM and due to DM increased with age, and the CMR levels were higher among men. Similarly, for both measures, the total ASMRW and YLL rates increased with age, and men had more quickly increasing rates. In addition, these rates were higher among people who died of causes associated with DM compared to those who died due to DM. These results indicate that individuals with concomitant DM, especially men, are more likely to experience higher mortality rates and YLL than people who died due to DM alone.

The mortality trend observed in our study is consistent with a previous study that analyzed 22 prospective cohorts in Asia from 1963 to 2006 and found a consistent increase in DM mortality, with higher all-cause mortality among women [21]. Another study that estimated DM mortality in different provinces also showed that the increase in age-standardized mortality among men from 2005 to 2020 was higher than among their female counterparts [22]. In contrast, our results are inconsistent with studies conducted in developed countries, which reported a decrease in DM mortality. For example, in the United States, all-cause mortality declined by 20% every 10 years, and specific-cause mortality due to DM complications also decreased from 1988 to 1994 and from 2010 to 2015 [18]. Such a decrease in DM mortality was also found in England [23] and Australia [24]. Such discrepancies in findings between our study and those from other developed regions may be attributed to differences in population aging and urbanization, as supported by our analysis of demographic and nondemographic factors. First, Shanghai has experienced rapid growth in the older population during the 21st century. Pudong has been classified as an aging society since 1982 (7.72% of the population was aged >65 years), an aged society since 2008 (14.21% aged >65 years), and a hyper-aged society since 2018 (21.81% aged >65 years; Multimedia Appendix 1, Figure S3) [25]. The accelerating process of population aging is accompanied by increases in the prevalence and mortality of DM with age, as well as increases in the number of more chronic complications, which are associated with adverse prognoses. Kirkman et al [26] showed that more than 25% of the population aged ≥65 years in the United States had DM, and that population aging drives the epidemic of DM. They also systematically analyzed the impact of population aging on people with DM and the recommended measures to address this issue. Second, as urbanization continues to progress (accompanied by economic and cultural development), people in Pudong have changed their dietary and lifestyle habits, resulting in substantial health impacts [27]. These changes have contributed to an increasing prevalence of metabolism-related diseases, such as obesity, metabolic syndrome, DM, hypertension, hyperlipidemia, and heart disease, which are more prevalent among regions undergoing urbanization and transition. Many studies have shown that the prevalence of DM is higher among more affluent and better educated populations, particularly in low-income countries [28,29]. In addition, as the pace of life speeds up, fewer people find time to engage in regular exercise, despite physical activity playing a crucial role in managing diabetes. Numerous studies have emphasized the importance of increased physical activity and fitness in alleviating DM, as well as calorie restriction and weight loss [30,31].

In addition, people aged ≥80 years exhibited the highest mortality and increase in YLL rate, probably due to their increased age. However, we also observed substantial mortality and increased YLL rates in people aged 30 to 44 years and 45 to 59 years that were associated with DM. These two age groups constitute a significant portion of the social workforce, and an increased disease burden in these groups exerts a significant adverse impact on economic development. Several potential explanations may account for these findings. First, patients with DM in these age groups may not have a comprehensive understanding of the severity of DM and its complications, resulting in low medication adherence and limited self-care behaviors. A study conducted in Ghana confirmed that patients aged 70 years and older were more likely to adhere to medication compared to those younger than 50 years [32]. Moreover, the decreasing YLL rate among deaths due to DM in people aged 70 to 79 years further supports this finding. Second, a younger age at diagnosis of type 2 DM is associated with higher mortality [33]. A previous study demonstrated that young DM patients exhibit worse glycemic control and face greater difficulty meeting glycohemoglobin and low-density lipoprotein cholesterol treatment goals [34]. Finally, people in these age ranges often shoulder the responsibility of supporting their households and have demanding work schedules, leaving limited time for complying with healthy lifestyle and medication regimens. As discussed in the previous section, urbanization has contributed to the increased burden of diseases.

In this study, we observed a high prevalence of multimorbidity among patients with DM, particularly in relation to cardiovascular disease, which includes hypertension, cerebrovascular disease, and ischemic heart disease. Hypertension emerged as the most prevalent comorbidity among deaths associated with DM. This finding aligns with the research conducted by Safar et al [35], who reported significantly higher cardiovascular risk in subjects with DM receiving treatment for hypertension, despite adequate glycemic and blood pressure control. Numerous studies have established that DM and cardiovascular disease have similar risk factors and pathogenic mechanisms, including a combination of genetic and metabolic factors, such as insulin resistance, contributing to their prevalence [36]. As mentioned earlier, hyperglycemia plays a pivotal role in the development of cardiovascular and cerebrovascular complications. DM stands as the leading risk factor for cardiovascular mortality due to its prominent involvement in cardiovascular pathogenesis [37]. Moreover, previous studies have shown that increased cancer incidence [38] and mortality are associated with DM [39]. Consistent with these findings, our study found a higher prevalence of various cancers among people who died of causes associated with DM. These cancers included liver cancer, colorectal cancer, malignant neoplasm of the pancreas, and stomach cancer. Patients with DM may be at increased risk of cancer due to shared risk factors, such as age, obesity, physical inactivity, and smoking. Therefore, the presence of multimorbidity of DM underscores the significant disease burden and the necessity of the management of glycemia and its associated complications, particularly cardiovascular disease and malignant tumors. Regular testing and follow-ups at community health centers should be prioritized to enhance overall care.

With the significant burden of DM, health policies play a crucial role in promoting education and support for the management of chronic disease. In 2005, Shanghai first piloted a community-based diabetes management policy and offered free health examinations for the elderly. Although we have not yet observed an inflection point in the mortality rate, we noted a stabilization of CMR and ASMRW among deaths due to DM in the past 5 years, following the revision and improvement of the policy (Multimedia Appendix 1, Figure S4). Furthermore, the Chinese government approved the Healthy China 2030 Strategy in 2016. In accordance with the national plan, Shanghai implemented its own plan to achieve the objectives in the Health Shanghai 2030 Planning Outline, which called for comprehensive interventions for health issues, lifelong health care, prevention and control of major diseases, and improvement of the health service system. Health management was emphasized in this plan and actively encouraged in community health centers and health management centers in district and municipal hospitals [40]. With improved individual socioeconomic status and health awareness, an increasing number of people are willing to undergo regular medical check-ups that screen for chronic diseases and malignant tumors, as well as undergo regular tests and follow-ups at community health centers, particularly for primary prevention of chronic diseases. With continual implementation of these policies, we anticipate an inflection point in DM mortality in the near future.

To our knowledge, this study is the first to analyze the disease burden of deaths associated with and due to DM in mainland China based on a large population (3.17 million people) and over a relatively long time frame (15 years.) The study area is representative and provides insights into the changing disease spectrum in transitioning regions. However, this study still has some limitations. First, a comprehensive life cycle analysis of the disease burden is currently unavailable, as we are still in the process of determining all causes of death, extrapolating relevant deaths during quality assessments, and refining the data. Second, although Shanghai is a transitional region in China, it is possible that its demographic and economic characteristics differ from those of other developing countries. Economic development will result in similar changes in lifestyle habits, making the data valuable as a reference. Third, our data lacked some details on DM, such as the clinical phenotype, duration, severity, treatment efficacy, and use of community health centers. Furthermore, other relevant factors, such as unhealthy lifestyle, low personal income, and adverse family circumstances, were also not captured in the data. Nonetheless, it is important to note that our study used complete and accurate public data from the government surveillance system, ensuring a high level of data quality.

### Conclusion

Population aging and multimorbidity have played essential roles in changing the burden of DM in an urbanizing and transitioning region. Young and middle-aged people have an increasing disease burden and should be given more attention. DM, an important disease associated with aging, remains an understudied and unaddressed medical threat in an aging society. Health management as an emerging method has important implications for alleviating the future burden of DM.

## Appendix

Multimedia Appendix 1 Additional material and data on deaths associated with and due to diabetes mellitus from 2005 to 2020 in Pudong, Shanghai, China.

## Abbreviations

AAPC: average annual percent change
APC: annual percent change
ASMRW: age-standardized mortality rate worldwide
CDC: Center for Disease Control and Prevention
CMR: crude mortality rate
DM: diabetes mellitus
*ICD-10*: *International Classification of Diseases Tenth version*
WHO: World Health Organization
YLL: years of life lost

## Data Availability

The data sets generated during and/or analyzed during this study are available from the corresponding author on reasonable request.
