# Supplementary material for: Patterns and Trends in Mortality Associated With and Due to Diabetes Mellitus in a Transitioning Region With 3.17 Million People: Observational Study
Source: JMIR Public Health Surveill. 2023 Sep 4;9:e43687. doi: 10.2196/43687 (PMC10507522; doi:10.2196/43687)

Table S1 | Age-specific mortality and the burden of deaths associated with and due to DM during 2005-2020.

Age group (years)	Deaths (N)	Proportion (%)	CMR (/10^5)	YLL (years)	YLL rate (/10^5)
**Deaths associated with DM**					
0-14	0	0.00	0.00	0.00	0.00
15-29	37	0.07	0.51	998.17	13.85
30-44	282	0.57	2.77	6678.27	65.62
45-59	3152	6.38	26.91	58742.07	501.41
60-69	7165	14.50	115.31	100335.50	1614.79
70-79	14374	29.09	439.24	133784.59	4088.18
⩾80	24404	49.39	1297.68	125400.22	6668.12
Total	49414	100.00	109.55	425938.83	944.27
**Deaths due to DM**					
0-14	0	0.00	0.00	0.00	0.00
15-29	24	0.15	0.33	649.20	9.01
30-44	153	0.99	1.50	3655.02	35.91
45-59	1051	6.78	8.97	19687.22	168.05
60-69	2125	13.70	34.20	29816.72	479.87
70-79	4327	27.89	132.22	40518.26	1238.15
⩾80	7832	50.49	416.46	40005.50	2127.28
Total	15512	100.00	34.39	134331.92	297.80

ASMRW, age-standardized mortality rate by Segi's world standard population; CMR, crude mortality rate; YLL, years of life lost.

Table S2 | The top 20 causes of death associated with diabetes mellitus and the top 20 comorbidities of patients who died due to diabetes mellitus.

Panel A:The top 20 causes of 49,414 deaths death associated with DM

Rank	ICD-10	Diseases(List of three-character categories)	Deaths(n)	Proportation(%)	ICD-10	Diseases(List of three-character categories)	Deaths of male(n)	Proportation(%)	ICD-10	Diseases(List of three-character categories)	Deaths of female(n)	Proportation(%)
1	E10-14	Diabetes mellitus	15512	31.39%	E10-14	Diabetes mellitus	7199	29.74%	E10-14	Diabetes mellitus	8313	32.98%
2	I20-25	Ischemic heart disease	9075	18.37%	I60-69	Cerebrovascular disease	4056	16.75%	I20-25	Ischemic heart disease	5082	20.16%
3	I60-69	Cerebrovascular disease	8695	17.60%	I20-25	Ischemic heart disease	3993	16.49%	I60-69	Cerebrovascular disease	4639	18.41%
4	C33-34	Lung cancer	2369	4.79%	C33-34	Lung cancer	1669	6.89%	J40-47	Chronic lower respiratory diseases	880	3.49%
5	J40-47	Chronic lower respiratory diseases	2339	4.73%	J40-47	Chronic lower respiratory diseases	1459	6.03%	C33-34	Lung cancer	700	2.78%
6	C22	Liver cancer	1130	2.29%	C22	Liver cancer	711	2.94%	C25	Malignant neoplasm of Pancreas	503	2.00%
7	C18-21	Colorectal Cancer	1055	2.14%	C18-21	Colorectal Cancer	582	2.40%	C18-21	Colorectal Cancer	473	1.88%
8	C25	Malignant neoplasm of Pancreas	1052	2.13%	C25	Malignant neoplasm of Pancreas	549	2.27%	C22	Liver cancer	419	1.66%
9	C16	Stomach cancer	887	1.80%	C16	Stomach cancer	530	2.19%	C16	Stomach cancer	357	1.42%
10	W00-19	Falls	582	1.18%	W00-19	Falls	256	1.06%	W00-19	Falls	326	1.29%
11	K70-77	Diseases of liver	446	0.90%	C61	Prostate	245	1.01%	C50	Malignant neoplasm of Breast	279	1.11%
12	C23-24	Malignant neoplasm of Gallbladder etc.	398	0.81%	K70-77	Diseases of liver	175	0.72%	K70-77	Diseases of liver	271	1.08%
13	B15-19	Coronary atherosclerotic heart disease	318	0.64%	B15-19	Coronary atherosclerotic heart disease	163	0.67%	C23-24	Malignant neoplasm of Gallbladder etc.	271	1.08%
14	K80-87	Disorders of gallbladder, biliary tract and pancreas	314	0.64%	C15	Malignant neoplasm of Oesophagu sV	132	0.55%	K80-87	Disorders of gallbladder, biliary tract and pancreas	205	0.81%
15	C50	Malignant neoplasm of Breast	281	0.57%	C67	Malignant neoplasm of Bladder	131	0.54%	B15-19	Coronary atherosclerotic heart disease	155	0.61%
16	I10-15	Hypertensive diseases	267	0.54%	I05-52	Heart disease	130	0.54%	I10-15	Hypertensive diseases	139	0.55%
17	I05-52	Heart disease	262	0.53%	I10-15	Hypertensive diseases	128	0.53%	I05-52	Heart disease	132	0.52%
18	C61	Prostate	245	0.50%	C23-24	Malignant neoplasm of Gallbladder etc.	127	0.52%	C70-72	Malignant neoplasm of Brain, central nervous system	119	0.47%
19	C70-72	Malignant neoplasm of Brain, central nervous system	209	0.42%	C81-85,C96	Malignant neoplasm of Lymphoma	114	0.47%	C91-95	Leukaemia	103	0.41%
20	C15	Malignant neoplasm of Oesophagu s	206	0.42%	K80-87	Disorders of gallbladder, biliary tract and pancreas	109	0.45%	C81-85,C96	Malignant neoplasm of Lymphoma	91	0.36%
	Total	Diseases(List of three-character categories)	49414	100.00%	Total	Diseases(List of three-character categories)	24210	100.00%	Total	Diseases(List of three-character categories)	25204	100.00%

Panel B: The top 20 causes of 15,512 deaths due to DM

Rnak	ICD-10	Diseases(List of three-character categories)	Deaths(n)	Proportation(%)	ICD-10	Disease(List of three-character categories)	Deaths of male(n)	Proportation(%)	ICD-10	Diseases(List of three-character categories)	Deaths of female(n)	Proportation(%)
	E10-14	Diabetes mellitus	15637		E10-14	Diabetes mellitus	7256		E10-14	Diabetes mellitus	8381	
1	I10-15	Hypertensive diseases	7671	23.69%	I10-15	Hypertensive diseases	3520	22.81%	I10-15	Hypertensive diseases	4151	24.48%
2	J95-99	Other diseases of the respiratory system	5254	16.23%	J95-99	Other diseases of the respiratory system	2627	17.03%	J95-99	Other diseases of the respiratory system	2627	15.49%
3	I60-69	Cerebrovascular disease	3913	12.08%	I60-69	Cerebrovascular disease	1869	12.11%	I60-69	Cerebrovascular disease	2044	12.05%
4	I20-25	Ischemic heart disease	3044	9.40%	I20-25	Ischemic heart disease	1343	8.70%	I20-25	Ischemic heart disease	1701	10.03%
5	I05-52	Heart disease	2714	8.38%	I05-52	Heart disease	1307	8.47%	I05-52	Heart disease	1407	8.30%
6	N17-19	Renal failure	2527	7.80%	N17-19	Renal failure	1290	8.36%	N17-19	Renal failure	1237	7.29%
7	J10-18	Chronic lower respiratory diseases	1391	4.30%	J10-18	Chronic lower respiratory diseases	740	4.80%	J10-18	Chronic lower respiratory diseases	651	3.84%
8	E70-90	Metabolic disorders	1111	3.43%	E70-90	Metabolic disorders	524	3.40%	E70-90	Metabolic disorders	587	3.46%
9	R50-69	General symptoms and signs	760	2.35%	R95-99	Ill-defined and unknown cause of mortality	362	2.35%	L80-99	Other disorder of the skin and subcutaneou s tissue	416	2.45%
10	R95-99	Ill-defined and unknown cause of mortality	714	2.20%	R50-69	General symptoms and signs	356	2.31%	R50-69	General symptoms and signs	404	2.38%
11	L80-99	Other disorder of the skin and subcutaneou s tissue	697	2.15%	L80-99	Other disorder of the skin and subcutaneou s tissue	281	1.82%	R95-99	Ill-defined and unknown cause of mortality	352	2.08%
12	K90-93	Other diseases of the digestive system	439	1.36%	J40-47	Chronic lower respiratory diseases	245	1.59%	F00-09	Organic, including symptomatic,mental disorders	215	1.27%
13	J40-47	Chronic lower respiratory diseases	416	1.28%	K90-93	Other diseases of the digestive system	227	1.47%	K90-93	Other diseases of the digestive system	212	1.25%
14	F00-09	Organic, including symptomatic,mental disorders	348	1.07%	A0-B99	Certain infectious and parasitic diseases	137	0.89%	J40-47	Chronic lower respiratory diseases	171	1.01%
15	A0-B99	Certain infectious and parasitic diseases	276	0.85%	F00-09	Organic, including symptomatic,mental disorders	133	0.86%	N30-39	Other diseases of the urinary system	155	0.91%
16	D60-64	Aplastic and other anaemias	268	0.83%	D60-64	Aplastic and other anaemias	115	0.75%	D60-64	Aplastic and other anaemias	153	0.90%
17	N30-39	Other diseases of the urinary system	242	0.75%	K70-77	Diseases of liver	107	0.69%	K80-87	Disorders of gallbladder, biliary tract and pancreas	141	0.83%
18	K70-77	Diseases of liver	225	0.69%	N30-39	Other diseases of the urinary system	87	0.56%	A0-B99	Certain infectious and parasitic diseases	139	0.82%
19	K80-87	Disorders of gallbladder, biliary tract and pancreas	219	0.68%	N40-51	Diseases of male genital organs	81	0.52%	K70-77	Diseases of liver	118	0.70%
20	G20-26	Extrapyramidal dyskinesia	152	0.47%	K80-87	Disorders of gallbladder, biliary tract and pancreas	78	0.51%	S70-79	Injury of hip and thigh	77	0.45%

Table S3 | The case number, CMR and ASMRW of deaths associated with DM according to sex during the period from 2005 to 2020 in the Shanghai Pudong, China.

Year	Male			Female			Total		
	Deaths (n)	CMR (/10^5)	ASMRW (/10^5)	Deaths (n)	CMR (/10^5)	ASMRW (/10^5)	Deaths (n)	CMR (/10^5)	ASMRW (/10^5)
2005	651	51.09	26.93	831	65.56	25.77	1482	58.31	26.20
2006	743	57.35	30.52	846	65.62	25.98	1589	61.47	27.99
2007	859	65.35	33.08	1049	80.09	30.00	1908	72.71	31.57
2008	892	66.89	31.90	1088	81.66	28.83	1980	74.27	30.30
2009	1078	79.78	36.63	1226	90.60	31.69	2304	85.19	34.09
2010	1094	79.97	35.80	1268	92.39	30.47	2362	86.19	32.97
2011	1236	89.41	38.41	1364	98.18	30.94	2600	93.81	34.54
2012	1408	100.91	42.15	1469	104.71	31.07	2877	102.82	36.36
2013	1438	102.11	41.95	1579	111.50	32.23	3017	106.81	37.02
2014	1553	108.85	42.12	1650	115.03	31.91	3203	111.95	36.86
2015	1750	120.94	45.95	1774	121.97	32.91	3524	121.45	39.21
2016	1827	124.69	45.89	1858	126.14	33.78	3685	125.42	39.67
2017	2112	142.39	50.98	2069	138.68	35.41	4181	140.53	42.91
2018	2226	148.24	51.90	2176	143.88	35.42	4402	146.05	43.60
2019	2619	172.04	57.76	2418	157.43	36.84	5037	164.71	46.96
2020	2724	176.46	57.98	2539	162.77	36.76	5263	169.58	47.22
Total	24210	107.54	43.84	24210	111.55	32.61	49414	109.55	38.01

ASMRW, age-standardized mortality rate by Segi's world standard population; CMR, crude mortality rate.

Table S4| The case number, CMR and ASMRW of deaths due to DM according to sex during the period from 2005 to 2020 in the Shanghai Pudong, China.

Year	Male			Female			Total		
	Deaths (n)	CMR (/10^5)	ASMRW (/10^5)	Deaths (n)	CMR (/10^5)	ASMRW (/10^5)	Deaths (n)	CMR (/10^5)	ASMRW (/10^5)
2005	243	19.07	10.02	394	31.08	11.79	637	25.06	10.96
2006	276	21.30	11.75	384	29.78	11.73	660	25.53	11.79
2007	280	21.30	10.66	410	31.30	11.85	690	26.29	11.33
2008	287	21.52	10.43	389	29.20	10.32	676	25.36	10.38
2009	311	23.02	10.81	428	31.63	10.64	739	27.33	10.87
2010	335	24.49	10.86	413	30.09	9.99	748	27.30	10.39
2011	355	25.68	11.46	466	33.54	10.64	821	29.62	11.13
2012	428	30.68	13.10	458	32.65	9.62	886	31.66	11.36
2013	419	29.75	12.74	512	36.15	10.29	931	32.96	11.53
2014	469	32.87	12.96	523	36.46	9.98	992	34.67	11.45
2015	552	38.15	15.02	584	40.15	10.69	1136	39.15	12.85
2016	567	38.70	14.55	615	41.75	11.39	1182	40.23	12.92
2017	636	42.88	15.33	665	44.57	11.43	1301	43.73	13.29
2018	610	40.62	14.29	666	44.04	10.86	1276	42.34	12.60
2019	731	48.02	16.29	705	45.90	10.63	1436	46.96	13.38
2020	700	45.35	14.91	701	44.94	9.98	1401	45.14	12.43
Total	7199	31.98	13.20	8313	36.79	10.79	15512	34.39	11.98

ASMRW, age-standardized mortality rate by Segi's world standard population; CMR, crude mortality rate.

Table S5 | The CMRs of age groups during the period from 2005 to 2020 in the Shanghai Pudong, China.

Year	0-14yrs	15-29yrs	30-44yrs	45-59yrs	60-69yrs	70-79yrs	⩾80 yrs
**Deaths associated with DM**							
2005	0	0.18	1.25	16.05	98.91	356.12	659.45
2006	0	0.55	3.14	16.35	103.05	356.17	740.21
2007	0	0.55	2.59	21.63	105.78	421.83	844.16
2008	0	0.19	1.40	20.80	99.07	394.53	894.47
2009	0	0.38	1.40	25.70	109.19	446.37	973.03
2010	0	0.78	2.60	22.22	104.55	426.99	1015.70
2011	0	0.81	2.53	22.32	101.34	451.81	1121.60
2012	0	0.43	3.39	26.13	95.90	470.30	1234.51
2013	0	0.92	1.55	29.75	102.72	472.32	1214.24
2014	0	0.00	3.67	27.39	108.03	444.35	1271.58
2015	0	0.98	2.41	28.13	117.28	469.00	1331.70
2016	0	1.28	2.82	35.88	113.30	428.43	1386.43
2017	0	0.81	2.74	33.31	124.82	452.10	1569.28
2018	0	0.28	3.93	34.65	130.95	469.07	1553.62
2019	0	0.00	4.50	37.17	134.03	466.80	1844.97
2020	0	0.00	3.44	39.04	140.75	460.36	1870.71
Total	0	0.51	2.77	26.90	115.51	438.88	1298.98
**Deaths due to DM**							
2005	0	0.00	0.89	6.80	36.14	150.67	305.89
2006	0	0.55	2.27	6.43	42.98	144.75	308.64
2007	0	0.37	1.38	7.78	33.11	153.64	313.51
2008	0	0.19	0.70	7.70	32.50	129.75	312.18
2009	0	0.19	0.88	8.31	33.65	131.97	334.74
2010	0	0.39	1.21	7.03	32.77	122.37	343.80
2011	0	0.61	1.35	7.69	35.89	129.84	355.56
2012	0	0.22	1.77	9.27	29.49	138.51	378.23
2013	0	0.69	1.24	8.69	28.20	140.20	392.19
2014	0	0.00	1.68	8.23	33.33	127.48	407.58
2015	0	0.74	2.11	9.56	34.14	143.75	441.97
2016	0	1.02	1.33	12.48	36.03	126.01	453.49
2017	0	0.27	1.01	11.74	36.10	133.89	501.16
2018	0	0.00	2.25	11.09	34.79	125.51	468.90
2019	0	0.00	2.05	12.44	33.86	122.01	549.41
2020	0	0.00	1.59	9.84	36.33	110.87	522.71
Total	0	0.33	1.50	8.97	34.26	132.12	416.88

CMR, crude mortality rate.

Table S6 | The YLL according to sex and age group during the period from 2005 to 2020 in the Shanghai Pudong, China.

Year	Male	Female	Total	0-14yrs	15-29yrs	30-44yrs	45-59yrs	60-69yrs	70-79yrs	≥80yrs
**Deaths associated with DM**										
2005	482.18	1193.16	836.75	0	4.96	55.39	474.03	2161.53	4929.88	3902.70
2006	552.39	630.36	591.28	0	14.83	73.37	310.18	1447.38	3402.71	3057.52
2007	634.71	755.34	694.92	0	14.84	61.54	408.76	1497.66	3973.05	3796.05
2008	622.59	745.67	684.10	0	4.96	32.73	398.07	1410.62	3666.47	4368.61
2009	754.17	832.48	793.35	0	10.28	33.99	491.35	1553.00	4169.14	5039.45
2010	733.44	822.01	777.79	0	21.55	60.35	419.26	1444.38	3952.09	5523.84
2011	809.28	844.36	826.87	0	21.69	60.32	417.27	1430.90	4149.63	6473.51
2012	910.37	876.00	893.14	0	11.62	80.34	493.75	1349.99	4301.43	7537.77
2013	930.27	923.38	926.81	0	24.85	36.14	553.72	1429.12	4362.15	7805.48
2014	970.60	938.86	954.69	0	0.00	88.62	506.48	1513.93	4074.21	8693.47
2015	1075.00	983.60	1029.18	0	26.58	57.74	517.15	1635.15	4329.84	9559.34
2016	1106.53	1018.13	1062.21	0	34.57	65.79	655.38	1580.98	3990.58	10035.96
2017	1243.31	1087.72	1165.29	0	21.96	66.58	615.90	1735.96	4251.52	11519.33
2018	1323.84	1113.27	1218.17	0	7.47	93.15	632.74	1824.09	4379.64	11482.10
2019	1477.49	1185.99	1331.09	0	0.00	105.97	686.42	1862.06	4377.36	13731.88
2020	1553.05	1230.02	1390.82	0	0.00	83.02	701.06	1991.94	4721.65	14012.19
Total	964.92	923.69	944.27	0	13.85	65.62	501.41	1614.79	4088.18	125400.22
**Deaths due to DM**										
2005	177.71	504.67	340.77	0	0.00	38.95	188.45	770.17	2006.49	2351.58
2006	211.79	285.81	248.71	0	14.83	53.47	122.84	594.05	1397.04	1649.42

2007	200.95	300.83	250.80	0	9.99	33.26	148.21	475.83	1454.13	1693.60
2008	200.31	268.62	234.45	0	4.96	16.64	147.66	460.84	1208.76	1671.13
2009	228.50	278.31	253.42	0	5.05	21.33	157.99	481.89	1247.31	1801.42
2010	220.61	262.56	241.62	0	10.98	28.38	130.40	453.38	1123.03	1758.87
2011	244.05	293.10	268.64	0	16.34	32.87	145.83	506.08	1195.52	1910.17
2012	293.46	270.01	281.70	0	5.91	42.94	175.52	418.56	1269.94	2009.50
2013	274.92	291.47	283.22	0	18.69	28.90	164.63	393.77	1302.21	2028.73
2014	295.54	291.98	293.75	0	0.00	40.35	153.79	464.13	1177.40	2103.83
2015	351.70	315.30	333.46	0	19.85	50.40	177.08	473.38	1342.79	2281.72
2016	348.01	339.98	343.99	0	27.69	31.45	229.70	501.77	1190.35	2285.71
2017	373.07	350.23	361.62	0	7.27	24.88	217.97	504.79	1263.19	2510.62
2018	362.39	339.17	350.74	0	0.00	53.58	202.75	485.56	1166.82	2299.54
2019	413.22	340.87	376.89	0	0.00	49.04	229.69	476.99	1140.53	2670.88
2020	397.05	330.92	363.84	0	0.00	38.71	176.97	513.12	1152.75	2526.93
Total	290.86	304.72	297.80	0	9.01	35.91	168.05	479.87	1238.15	2127.28

YLL rate, the rate of years of life lost.

Table S7| The proportion of deaths according to age group during the period from 2005 to 2020 in the Shanghai Pudong, China.

year	0-14yrs	15-29yrs	30-44yrs	45-59yrs	60-69yrs	70-79yrs	⩾80yrs
**Deaths associated with DM**							
2005	0.00	0.30	0.47	7.96	14.04	43.86	33.60
2006	0.00	0.82	1.13	7.68	14.03	41.35	35.62
2007	0.00	0.74	0.79	8.60	12.89	41.30	36.27
2008	0.00	0.29	0.40	8.18	12.78	37.78	40.81
2009	0.00	0.54	0.35	8.85	13.24	36.85	40.63
2010	0.00	1.33	0.64	7.49	13.51	34.42	43.78
2011	0.00	1.36	0.58	6.81	12.92	33.19	46.35
2012	0.00	0.74	0.73	7.06	12.10	31.04	49.01
2013	0.00	1.37	0.33	7.49	13.52	29.37	49.15
2014	0.00	0.00	0.75	6.34	14.67	26.23	52.01
2015	0.00	1.21	0.45	5.76	15.69	25.74	52.24
2016	0.00	1.45	0.52	6.87	15.36	23.53	53.60
2017	0.00	0.82	0.45	5.43	15.71	23.58	54.75
2018	0.00	0.27	0.64	5.18	16.33	25.22	52.61
2019	0.00	0.00	0.66	4.74	14.93	24.00	55.67
2020	0.00	0.00	0.49	4.67	15.24	24.85	54.74
Total	0.00	0.66	0.57	6.38	14.50	29.09	49.39
**Deaths due to DM**							
2005	0.00	0.00	0.78	7.85	11.93	43.17	36.26
2006	0.00	0.82	1.97	7.27	14.09	40.45	35.76
2007	0.00	0.50	1.16	8.55	11.16	41.59	37.25
2008	0.00	0.29	0.59	8.88	12.28	36.39	41.72
2009	0.00	0.27	0.68	8.93	12.72	33.96	43.57
2010	0.00	0.66	0.94	7.49	13.37	31.15	46.79
2011	0.00	1.02	0.97	7.43	14.49	30.21	46.53
2012	0.00	0.37	1.24	8.13	12.08	29.68	48.76
2013	0.00	1.02	0.86	7.09	12.03	28.25	51.45
2014	0.00	0.00	1.11	6.15	14.62	24.29	53.83
2015	0.00	0.91	1.23	6.07	14.17	24.47	53.79
2016	0.00	1.16	0.76	7.45	15.23	21.57	54.65
2017	0.00	0.27	0.54	6.15	14.60	22.44	56.19
2018	0.00	0.00	1.25	5.72	14.97	23.28	54.78
2019	0.00	0.00	1.04	5.57	13.23	22.01	58.15
2020	0.00	0.00	0.86	4.43	14.78	22.48	57.46
Total	0.00	0.43	0.99	6.78	13.70	27.89	50.49

Table S8 | The increase in CMRs caused by demographic and non-demographic factors during the period from 2006 to 2020 in the Shanghai Pudong, China.

year	non- demographic			demographic		
	Total	Male	Female	Total	Male	Female
**Deaths associated with DM**						
2006	3.29	6.33	0.23	-0.13	-0.08	-0.17
2007	12.55	12.30	12.80	1.85	1.95	1.74
2008	10.93	10.56	11.30	5.04	5.25	4.81
2009	19.44	20.58	18.23	7.45	8.11	6.81
2010	17.31	17.56	16.98	10.57	11.32	9.85
2011	23.22	24.96	21.34	12.29	13.36	11.28
2012	29.44	33.32	25.33	15.07	16.51	13.82
2013	30.52	32.00	28.83	17.99	19.01	17.11
2014	31.87	34.43	29.07	21.77	23.33	20.40
2015	38.18	42.62	33.41	24.96	27.22	23.00
2016	39.49	43.68	34.87	27.63	29.91	25.71
2017	49.68	55.28	43.57	32.55	36.02	29.55
2018	52.13	58.31	45.40	35.62	38.84	32.93
2019	64.56	74.49	53.77	41.84	46.46	38.11
2020	65.91	75.88	55.01	45.36	49.49	42.20
**Deaths due to DM**						
2006	0.53	2.26	-1.21	-0.06	-0.03	-0.09
2007	0.50	1.53	-0.52	0.73	0.70	0.74
2008	-1.63	0.59	-3.81	1.92	1.86	1.92
2009	-0.53	1.32	-2.31	2.80	2.63	2.85
2010	-1.79	1.43	-4.93	4.02	3.99	3.94
2011	0.07	2.27	-2.03	4.49	4.34	4.49
2012	1.26	6.19	-3.60	5.34	5.41	5.17
2013	1.61	4.61	-1.29	6.29	6.07	6.36
2014	1.88	5.96	-2.07	7.73	7.85	7.45
2015	5.25	10.12	0.49	8.84	8.95	8.58
2016	5.31	9.50	1.26	9.85	10.13	9.41
2017	7.39	11.94	2.97	11.27	11.87	10.52
2018	5.42	9.50	1.49	11.86	12.05	11.47
2019	8.30	14.76	1.85	13.59	14.19	12.97
2020	5.59	11.41	-0.15	14.49	14.86	14.01

CMR, crude mortality rate.

FIGURE S1 | The proportion of deaths associated with and due to diabetes mellitus out of all deaths during the period from 2005 to 2020 in Shanghai Pudong, China. A: The proportion of DM-associated deaths out of all deaths. B: The proportion of DM-caused deaths out of DM-associated deaths. C: The proportion of DM-caused deaths out of all deaths.

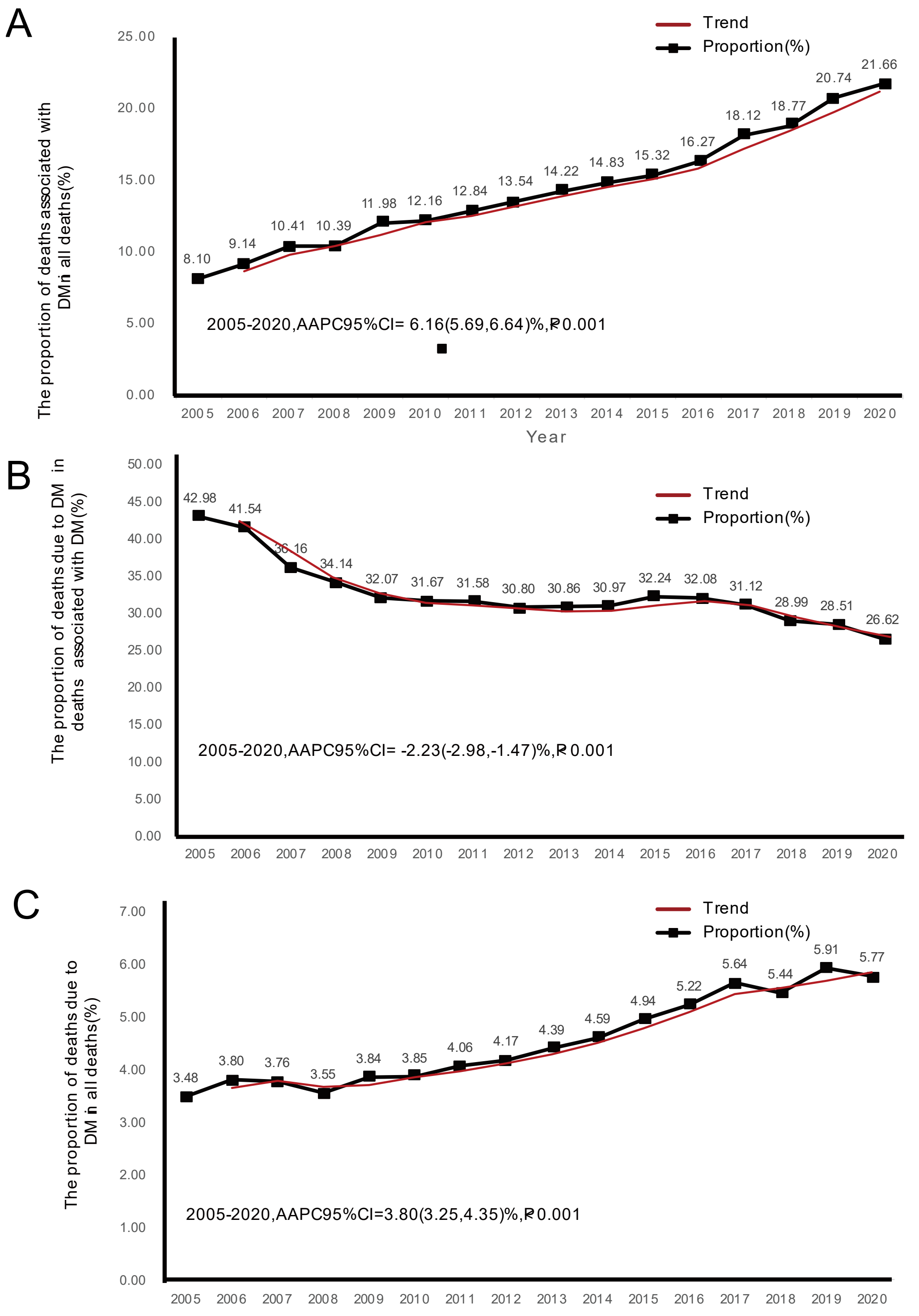

FIGURE S2 | The mortality rates caused by demographic and non-demographic factors during the period from 2005 to 2020 in the Shanghai Pudong, China. A: The increased mortality rates caused by demographic and non-demographic factors according to age group in people who died of causes associated with diabetes mellitus. B: The increased mortality rates caused by demographic and non-demographic factors according to age group in people who died due to diabetes mellitus. C: The trends of mortality rates caused by demographic and non-demographic factors according to age group in people who died of causes associated with and due to diabetes mellitus.

AAPC, average annual percent change; CI, confidence interval.

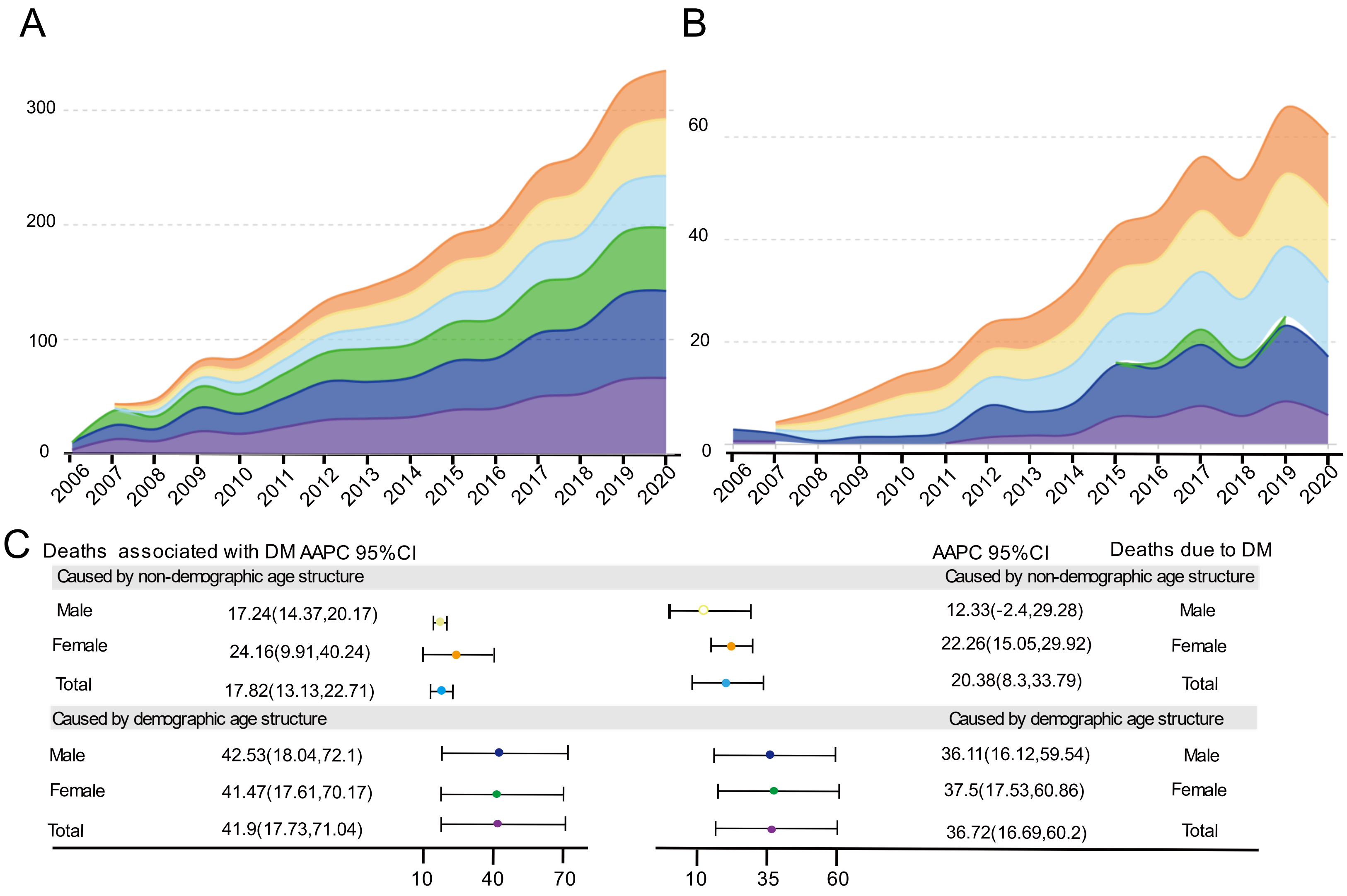
A
B
300
200
100
0
60
40
20
2006
2007
2008
2009
2010
2011
2012
2013
2014
2015
2016
2017
2018
2019
2020
C
Deaths associated with DM AAPC 95%CI
AAPC 95%CI
Deaths due to DM
Caused by non-demographic age structure
Male 17.24(14.37,20.17)
Female 24.16(9.91,40.24)
Total 17.82(13.13,22.71)
12.33(-2.4,29.28) Male
22.26(15.05,29.92) Female
20.38(8.3,33.79) Total
Caused by demographic age structure
Male 42.53(18.04,72.1)
Female 41.47(17.61,70.17)
Total 41.9(17.73,71.04)
36.11(16.12,59.54) Male
37.5(17.53,60.86) Female
36.72(16.69,60.2) Total
10
40
70
10
35
60

FIGURE S3 | The proportion of people aged ≥65 years during the period from 2005 to 2020 in the Shanghai Pudong, China.

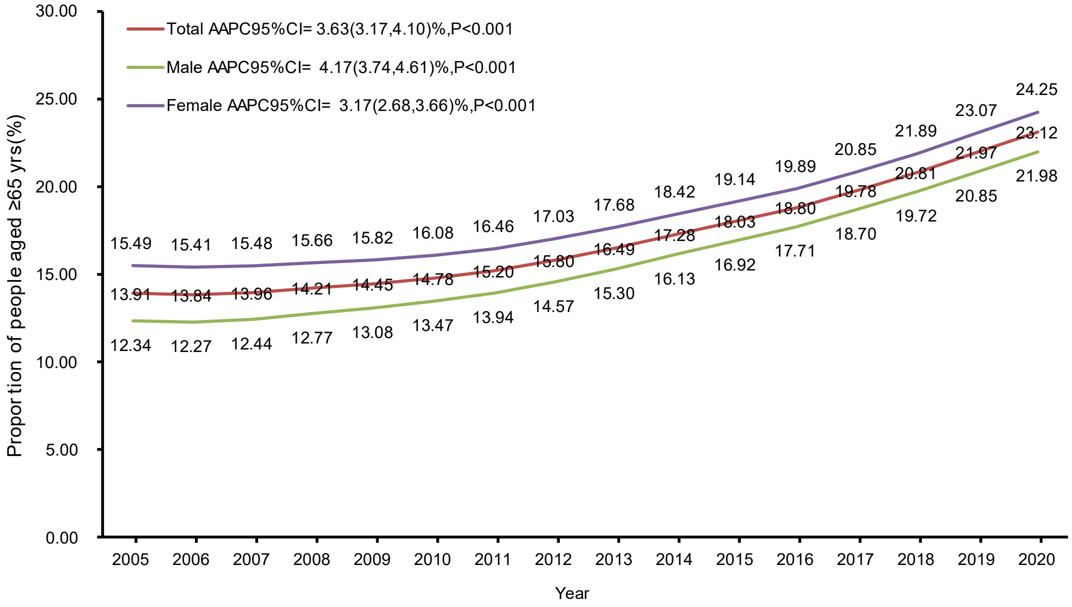

FIGURE S4 | The community management of diabetes mellitus in community health centers in the Shanghai Pudong, China.

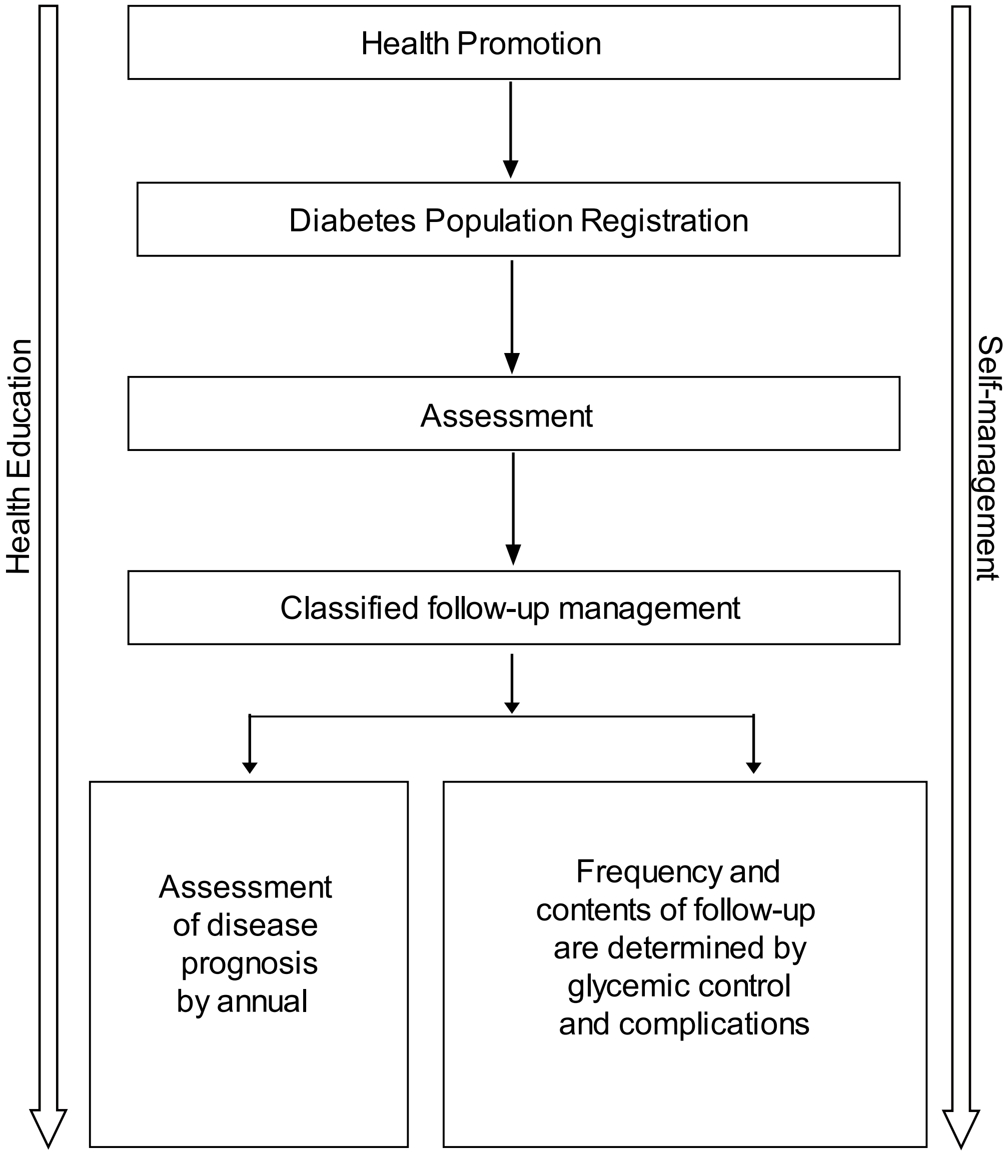

Supplement: Multimedia Appendix 1 [file publichealth_v9i1e43687_app1.pdf]
